# Line Tension and Stability of Domains in Cell-Adhesion Zones Mediated by Long and Short Receptor-Ligand Complexes

**DOI:** 10.1371/journal.pone.0023284

**Published:** 2011-08-17

**Authors:** Heinrich Krobath, Bartosz Różycki, Reinhard Lipowsky, Thomas R. Weikl

**Affiliations:** Max Planck Institute of Colloids and Interfaces, Department of Theory and Bio-Systems, Potsdam, Germany; German Cancer Research Center, Germany

## Abstract

Submicron scale domains of membrane-anchored receptors play an important role in cell signaling. Central questions concern the stability of these microdomains, and the mechanisms leading to the domain formation. In immune-cell adhesion zones, microdomains of short receptor-ligand complexes form next to domains of significantly longer receptor-ligand complexes. The length mismatch between the receptor-ligand complexes leads to membrane deformations and has been suggested as a possible cause of the domain formation. The domain formation is a nucleation and growth process that depends on the line tension and free energy of the domains. Using a combination of analytical calculations and Monte Carlo simulations, we derive here general expressions for the line tension between domains of long and short receptor-ligand complexes and for the adhesion free energy of the domains. We argue that the length mismatch of receptor-ligand complexes alone is sufficient to drive the domain formation, and obtain submicron-scale minimum sizes for stable domains that are consistent with the domain sizes observed during immune-cell adhesion.

## Introduction

In the past years, microdomains of proteins in cell membranes have emerged as a central aspect of cell signaling [1]–[4]. The activation of T cells, for example, is initiated by submicron-scale domains of T cell receptors (TCRs) [1], [5]–[7]. The TCRs recognize foreign peptides presented by MHC ligands (MHCpeptide) in an apposing cell membrane. During T-cell adhesion, domains of TCR-MHCpeptide form within seconds in the adhesion zone [8], [9].

Several mechanisms have been proposed for the formation of TCR-MHCpeptide domains during T-cell adhesion. These mechanisms are based on the actin cytoskeleton [10], [11], enhanced cis-interactions between TCRs due to conformational changes after binding [1], pre-clustering of TCRs prior to adhesion [12], [13], and the length difference between the TCR-MHCpeptide complexes and other receptor-ligand complexes and proteins in the T-cell adhesion zone [14]–[22]. We argue here that the length differences between TCR-MHCpeptide complexes and other complexes alone can account for the formation of clusters and domains during T-cell adhesion. The TCR-MHCpeptide complex has a length of about 13 nm [23]–[25], while complexes between the integrin LFA-1 and its ligand ICAM-1 have a length around 40 nm [10]. This length mismatch induces a membrane-mediated repulsion between different complexes in the T-cell contact zone because the membranes have to bend to compensate the mismatch, which costs bending energy. Beyond certain threshold or critical concentrations of the receptors and ligands, the membrane-mediated repulsion leads to a segregation of TCR-MHCpeptide and integrin complexes into domains enriched in these complexes.

In previous work, we have derived general expressions for the critical receptor and ligand concentrations required for domain formation. These general expressions depend on the length mismatch between the receptor-ligand complexes and on the bending rigidity of the membranes [26], [27]. We have also found that large, repulsive glycoproteins and additional complexes with a length close to the TCR-MHCpeptide complex, such as the CD2–CD48 complex [25], increase the tendency for domain formation [26].

In this article, we determine the free energy and stability of clusters and domains of long and short receptor-ligand complexes. Our main results are general expressions for the line tension and adhesion free energy of the domains in terms of the concentrations and affinities of the receptors and ligands as well as the length mismatch of the receptor-ligand complexes. These general expressions fully include the effects of membrane shape fluctuations and the translational entropy of the receptors and ligands, and depend only on experimentally accessible quantities. Our expressions lead to estimates for the minimal size of stable TCR-MHCpeptide microdomains that are consistent with the submicron-scale sizes observed during T-cell adhesion.

## Methods

### Membrane conformations, interactions and elasticity

To describe the conformations of the two apposing membranes in a cell adhesion zone, we divide these membranes into small patches. Each patch can contain a single receptor or ligand molecule [28]–[30]. A receptor binds to a ligand molecule if the ligand is located in the membrane patch apposing the receptor, and if the separation 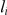 of the two membrane patches is close to the length of the receptor-ligand complex. The mobile receptor and ligand molecules diffuse by ‘hopping’ from patch to patch, and the thermal fluctuations of the membranes are reflected in variations of the separation of apposing membrane patches.

The energy of a membrane conformation
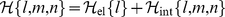
(1)is the sum of the elastic energy 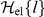 of the membranes and the interaction energy 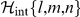 of the receptors and ligands. For a membrane with two types of receptors 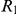 and 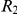 that bind to the ligands 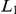 and 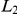 in the apposing membrane, the interaction energy is [27], [31]

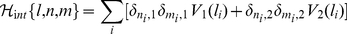
(2)Here, the occupation number 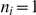, 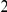, or 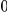 indicates whether a receptor 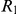, a receptor 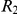, or no receptor is present in patch 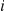 of the cell membrane in the contact zone, while 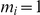, 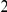, or 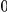 indicates whether a ligand 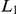, a ligand 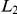, or no ligand is present in the apposing membrane patch 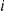. The Kronecker symbol 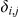 equals 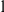 for 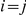 and is equal to 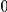 for 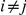. The potential 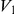 thus describes the interaction of a receptor 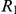 with a ligand 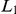, and the potential 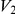 the interaction between 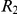 and 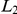. For simplicity, 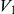 and 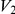 are taken to be square-well potentials
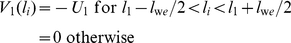
(3)and
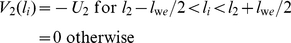
(4)with binding energies 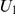 and 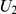 and equilibrium lengths 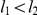 of the complexes 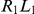 and 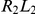. We have assumed here that the two complexes have the same binding width 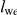.

The rigidity-dominated elastic energy of the membranes has the form [28], [30]

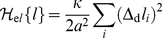
(5)where 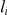 is the local separation of the apposing membrane patches 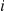. The elastic energy depends on the mean curvature 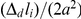 of the separation field 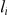 with the discretized Laplacian 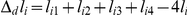. Here, 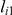 to 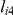 are the membrane separations at the four nearest-neighbor patches of membrane patch 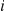 on the quadratic array of patches. The linear size 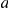 of the membrane patches is chosen to be around 5 nm to include the whole spectrum of bending deformations of the lipid membranes [32]. The effective bending rigidity of the two membranes with rigidities 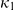 and 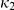 is 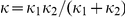. If one of the membranes, e.g. membrane 2, is a planar supported membrane, the effective bending rigidity 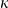 equals the rigidity 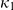 of the apposing membrane since the rigidity 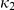 of the supported membrane is taken to be much larger than 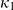.

### Effective adhesion potential of the membranes

**Figure 1:**
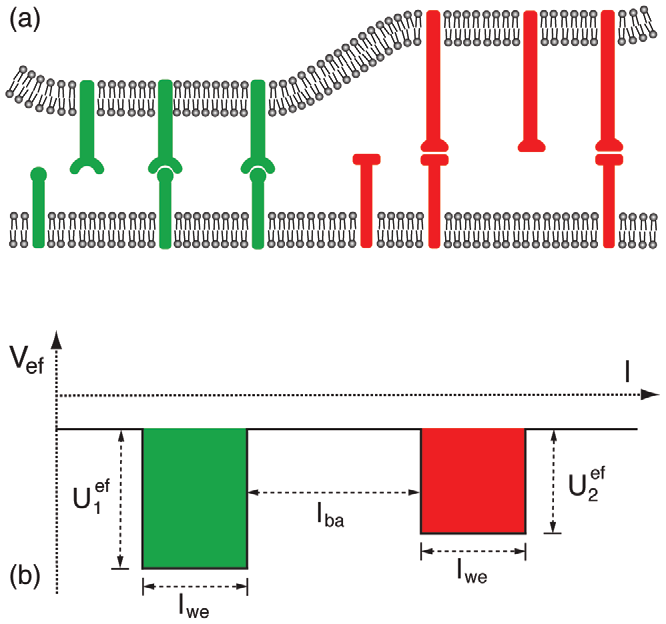
Effective adhesion potential. (a) Two membranes interacting *via* long (red) and short (green) receptor-ligand complexes. The length mismatch of the complexes causes membrane deformations, which cost bending energy and lead to a membrane-mediated repulsion between different receptor-ligand complexes. - (b) The attractive interactions between the two types of receptors and ligands lead to an effective double-well adhesion potential 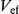 of the membranes. The potential well 1 at small membrane separations 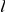 reflects the interactions of the short receptor-ligand complexes, and the potential well 2 at larger membrane separations the interactions of the long receptor-ligand complexes. The depths 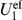 and 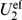 of the two potential wells depend both on the concentrations and on the binding energies of the two types of receptors and ligands, see eqs. (7) and (8).

The equilibrium proporties of the membranes can be determined from the free energy 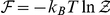 where 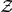 is the partition function. The partition function 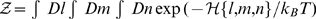 is the integral over all membrane conformations, with each conformation weighted by its Boltzmann factor. In our model, the integration over the distributions 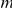 and 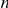 of receptors and ligands can be performed exactly [27], which leads to 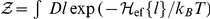 with the effective conformational energy
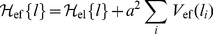
(6)For long and short receptors and ligands with interaction energy (2), the effective potential is a double-well potential (see fig. 1(b)). The two wells of this potential are centered around the lengths 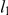 and 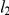 of the complexes 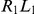 and 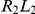, and the width 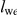 of these wells is equal to the binding width of the two complexes. The depths 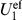 and 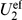 of the wells depend both on the concentrations and on the binding affinities of the receptors and ligands. The typical concentrations of receptors and ligands in cell adhesion zones are much smaller than the maximal concentration 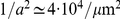 in our model. For these small concentrations, we obtain
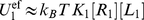
(7)

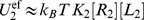
(8)where 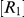, 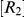, 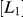 and 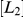 are the area concentrations of unbound receptors and ligands, and 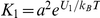 and 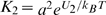 are the binding constants for receptors and ligands within the appropriate binding ranges [26], [27]. The summation over the degrees of freedom 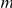 and 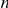 of the receptors and ligands thus ‘maps’ the problem of two membranes interacting *via* long and short receptor-ligand complexes to the problem of a membrane with effective rigidity 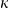 in an effective double-well potential.

The effective potential can be generalized to cases with more than two receptor-ligand complexes, or with additional repulsive molecules [26]. For T cells adhering to antigen-presenting cells, for example, a third important receptor-ligand complex is the CD2–CD48 complex, which has about the same length as the TCR-MHCpeptide complex. In this case, the well depth 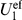 in the effective double-well potential depends on the concentrations and binding constants of TCR and MHCp as well as CD2 and CD48 [26]. In addition, complexes between TCRs and self MHCpeptide molecules, besides foreign MHCpeptides, can contribute to this well depth [26].

The effective potential helps to determine and illustrate the equilibrium behavior. If the two wells of the effective potential are relatively shallow, thermal membrane fluctuations can easily drive membrane segments to cross from one well to the other. If the two wells are deep, the crossing of membrane segments from one well to the other well is impeded by the potential barrier of width 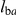 between the wells (see fig. 1). Beyond a critical depth of the potential wells, the potential barrier leads to the formation of large membrane domains that are predominantly bound in well 1 or well 2. Within each domain, the adhesion of the membranes is predominantly mediated either by the receptor-ligand complexes 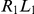 or by the complexes 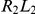, which leads to different concentrations 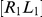 and 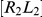 of these complexes in the different domains. However, the equilibrium concentrations 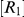, 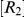, 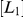, and 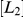 of unbound receptors and ligands are identical in the different domains since these receptors and ligands are free to diffuse between the domains. Therefore, the effective potential is also identical in the different domains (see eqs. (7) and (8)). In general, the diffusion of individual receptors and ligands is fast compared to the domain formation [20].

We have previously found that the critical potential depth for domain formation is
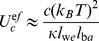
(9)with the prefactor 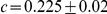 determined from Monte Carlo simulations [27]. Domain formation or, in other words, segregation of the complexes 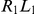 and 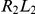 can only occur if the effective potential depths 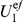 and 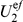 exceed the critical potential depth 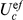. The critical potential depth depends on the temperature 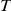 and the bending rigidity 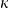 as well as on the width 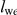 and separation 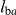 of the two potential wells. In deriving eq. (9), we have neglected direct membrane-membrane contacts, which is reasonable for typical concentrations and lengths of receptor-ligand complexes in cell adhesion zones since the thermal membrane roughness is smaller than the lengths of the receptor-ligand complexes for these concentrations and lengths [27], [33].

In this article, we determine how the adhesion free energy and line tension of the domains depends on the depths as well as on the width 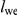 and separation 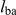 of the two wells. The starting point for our calculations and simulations is the effective conformational energy (6) with the double-well potential 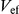 shown in fig. 1(b).

### Effective parameters and Monte Carlo simulations

We use a combination of Monte Carlo simulations and scaling arguments to determine the free energy difference and line tension of membrane domains that are bound in the two potential wells of the effective potential 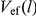. To reduce the number of parameters, we use the rescaled separation field 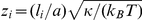 in the simulations. The effective conformational energy (6) then has the form 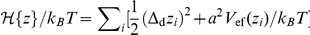 where 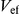 is the effective potential shown in fig. 1(b). The four parameters of the Monte Carlo simulations are the rescaled width and separation
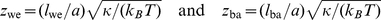
(10)of the potential wells, and the dimensionless well depths
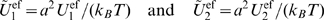
(11)


A scaling analysis (see Appendix S1) indicates that there are only three independent parameters if the lateral correlation length of the membranes is much larger than the linear size 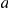 of the discrete membrane patches, which is the case if the membranes are only weakly bound in the potential wells. These three parameters are the rescaled well depths
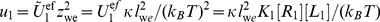
(12)

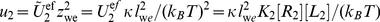
(13)and the ratio
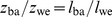
(14)of the separation and width of the potential wells. From eq. (9), we obtain the rescaled critical potential depth
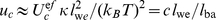
(15)with 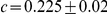, which depends only on the ratio of these two characteristic lengths of the double-well potential.

In the Monte Carlo simulations, we attempt local Monte Carlo moves in which the rescaled separation 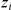 of the membrane patch 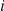 is shifted to a new value 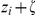 where 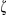 is a random number between 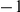 and 1. Following the standard Metropolis criterion [34], a local move is always accepted if the change 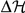 in conformational energy is negative, and accepted with the probability 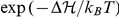 for 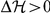. We perform simulations with up to 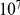 attempted local moves per patch 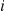 and membrane sizes up to 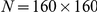 patches. The membrane size is always chosen to be much larger than the lateral correlation length of the membranes, since thermodynamic averages of membrane quantities then do not depend on the finite size of the membranes. Further details of our Monte Carlo simulations are described in ref. [30].

## Results

### Adhesion free energy of receptor-ligand domains

The free energy of domains of long or short receptor-ligand complexes can be determined from the effective double-well adhesion potential of the membranes (see fig. 1). We consider first a domain of short receptor-ligand complexes, i.e. a domain bound in well 1 of the effective adhesion potential. The free energy per area of this membrane domain is (see Appendix S2)
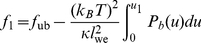
(16)where 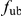 is the free energy per area of the unbound membrane, and 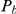 is the area fraction of the membrane domain bound in the well. The rescaled well depth 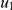 depends on the concentrations and affinity of the receptors and ligands, on the effective bending rigidity 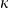 of the membranes, and on the width 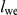 of the well (see eq. (12)). Similarly, the free energy per area of a domain of long receptor-ligand complexes, i.e. of a domain bound in well 2 of the effective potential, can be written as
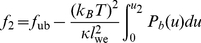
(17)For equal widths 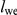 of the two potential wells, the free energy difference per area between domains bound in well 1 and well 2 is then
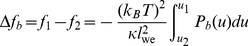
(18)


**Figure 2:**
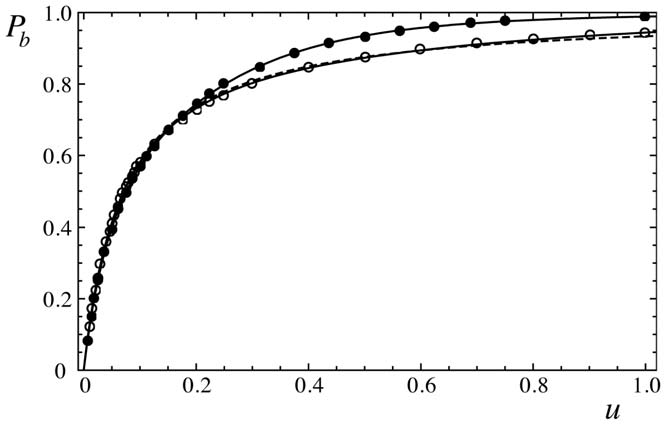
Fraction 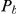 of membrane patches inside a single well as a function of the rescaled depth 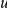 of the well. The data points are from Monte Carlo simulations with the rescaled well widths 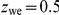 (filled circles) and 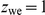 (open circles). The full lines result from fits of the three-parameter function 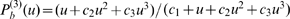 with 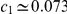, 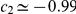, and 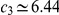 for 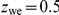 and 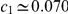, 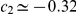, and 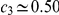 for 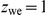. The dashed line results from a fit of eq. (19) with 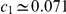 to the data points for 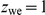 and 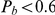 (see [33] for details).

The function 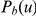 is linear in the rescaled well depth 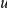 for small values of 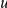, and attains the limiting value of 1 for large values of 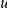 at which the membrane domain is essentially fully bound in the well [29], [33]. The precise form of this function can be easily determined from Monte Carlo simulations of a membrane bound in a single well (see fig. 2). To derive a general analytical expression for the free energy difference 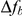, we consider here the single-parameter fit [33]

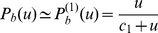
(19)with 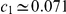 for the Monte Carlo data at the rescaled well width 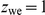. For 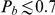, the single-parameter function 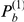 coincides with overall more precise three-parameter functions 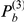 at 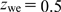 and 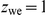 (see fig. 2). With eq. (19), we obtain the general expression
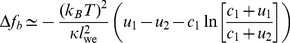
(20)for the adhesion free energy difference between domains bound in well 1 and well 2 of the effective adhesion potential shown in fig. 1(b).

### Classical nucleation theory of domain formation

We use classical nucleation theory to determine the line tension between domains of long and short receptor-ligand complexes. Equilibrium properties of the membranes, such as the line tension between the domains of receptor-ligand complexes, can be obtained from the effective double-well adhesion potential of the membranes shown in fig. 1(b). We consider now a circular membrane domain of radius 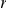 that is bound in well 1 of the effective adhesion potential, surrounded by a large domain bound in well 2. We assume that the rescaled depths of the two potential wells are beyond the critical depth (15), with 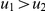. In classical nucleation theory, the excess free energy of the circular domain is
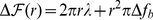
(21)where 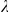 is the line tension of the domain boundary, and 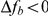 is the free energy difference per area between the two domains. The excess free energy has a maximum at the critical radius
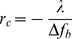
(22)which follows from 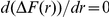. For radii 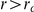, the circular domain grows into a stable domain since the excess free energy decreases with increasing 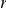. For radii 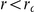, the circular domain is instable and shrinks since the excess free energy decreases with decreasing 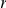. Using eq. (22), we will determine the line tension 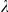 from the free energy differences 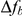 and the critical radii 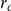 obtained from Monte Carlo simulations.

### Critical domains sizes from Monte Carlo simulations

**Figure 3:**
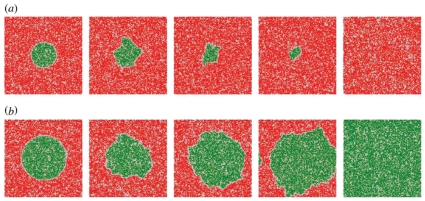
Stability of adhesion domains. (a) and (b): Time sequences of Monte Carlo snapshots of a membrane in the effective double-well potential of fig. 1(b) with rescaled depths 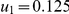 and 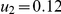, rescaled width 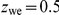 and rescaled separation 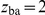. Membrane patches bound in well 1 are indicated in green, and membrane patches bound in well 2 are red. In (a), the initial radius of the green domain bound in well 1 of the effective potential is below the critical radius for domain stability. Therefore, the domain shrinks and finally vanishes in the simulations. The shapshots are taken at times 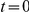, 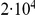, 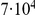, 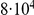, and 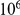 Monte Carlo steps per patch. In (b), the initial radius of the green domain is above the critical radius. The domain thus increases until the whole membrane is bound in the deeper potential well 1. The shapshots are taken at times 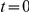, 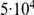, 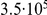, 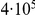, and 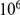 Monte Carlo steps per patch.

We determine the critical radii of domain nucleation from Monte Carlo simulations. The simulations start from pre-equilibrated initial conformations with a circular nucleus of radius 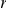 bound in the deeper well 1, surrounded by a membrane domain bound in well 2 (see fig. 3). The pre-equilibration ensures (i) that the circular nucleus contains the expected fraction 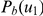 of membrane patches bound in well 1, (ii) that the surrounding domain contains a fraction 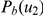 of membrane patches bound in well 2, and (iii) that the domain boundary is relaxed. To create a pre-equilibrated initial conformation, we ‘cut out’ a circular domain of radius 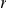 from a Monte Carlo simulation of a membrane that only ‘feels’ well 1, ‘freeze’ this domain, and place it into a membrane that only ‘feels’ well 2. The domain boundary is then relaxed by a simulation in which the nucleus remains ‘frozen’ (no Monte Carlo moves inside the nucleus), and in which the surrounding membrane continues to ‘feel’ only well 2.

**Figure 4:**
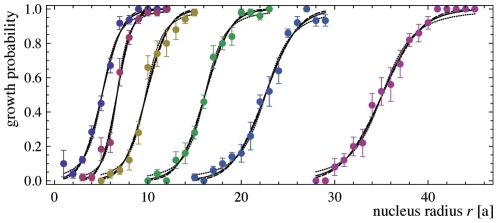
Growth probability of a circular nucleus as a function of the nucleus radius 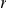 in units of the linear size 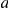 of the membrane patches. The initial nucleus is bound in well 1 of the effective adhesion potential shown in fig. 1(b), and the surrounding membrane is bound in well 2 (see Monte Carlo snapshots in fig. 3). The six curves are from simulations with the rescaled depths 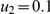, 0.105, 0.11, 0.115, 0.1175, and 0.12 of well 2 (from left to right). In all simulations, the rescaled depth of well 1 is 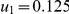, and the rescaled separation and width of the wells are 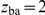 and 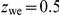. Each data point was obtained from averaging over 30 simulations. To extract the critical radius from a curve, we fit the curve with three different fit functions and determine the three radii 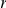 at which these fit functions attain the value 0.5. The critical radius 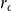 is defined as the average of these three radii. For the six curves, we obtain the values 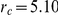, 6.70, 9.81, 16.12, 22.58, and 35.08 of the critical radius. The three fit functions are 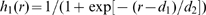 (full lines), 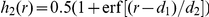 (dashed lines), and 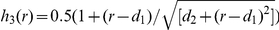 (dotted lines).

Nuclei with a radius 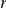 smaller than the critical radius 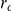 tend to shrink, while nuclei with a radius larger than 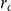 tend to grow (see fig. 3). To quantify this tendency of the nuclei to grow or shrink, we perform 30 simulations for each nucleus size, and determine the fraction of the simulations in which the nucleus grows. These growth fractions are displayed in fig. 4 for simulations with the rescaled depth 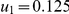 of well 1 and rescaled depths between 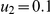 and 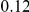 for well 2. After data smoothening, the critical radius 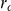 is defined as the radius at which the smoothed growth fractions have the value 0.5 (see caption of fig. 4 for details).

**Figure 5:**
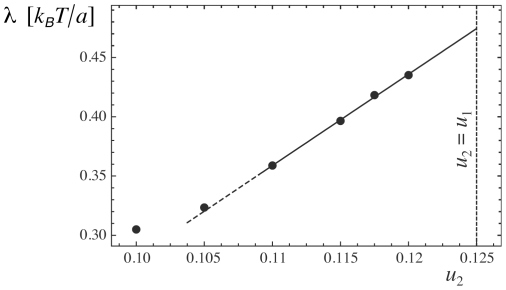
Line tension extrapolation. Line tension 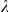 as function of the rescaled depth 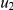 of well 2 for the rescaled depth 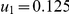 of well 1. The six data points result from the six values of the critical radius 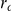 determined in fig. 4. The line tension 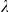 is obtained from the critical radii as 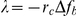 (see eq. (22)), with the free energy difference 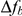 calculated from eq. (18) with the function 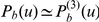 given in Appendix S2 and in the caption of fig. 2. Linear extrapolation of the four right data points leads to the estimated value 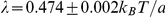 for the line tension of the symmetric double-well potential with rescaled depth 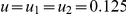 and rescaled separation 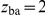 and 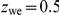 of the wells.

According to eq. (22), the line tension now follows as 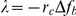 from the critical radii 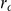 and the free energy differences 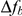, which are calculated from eq. (18) with 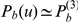 where 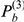 is the three-parameter function at the rescaled well width 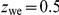 shown in fig. 2. The resulting values for the line tension are shown in fig. 5. The line tension increases with the rescaled depth 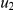 of well 2 since the potential barrier increases (see fig. 1(b)). The line tension for the symmetric double-well potential with well equal depths 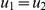 can be obtained from extrapolation (see fig. 5).

### Line tension between domains of long and short receptor-ligand complexes

**Figure 6:**
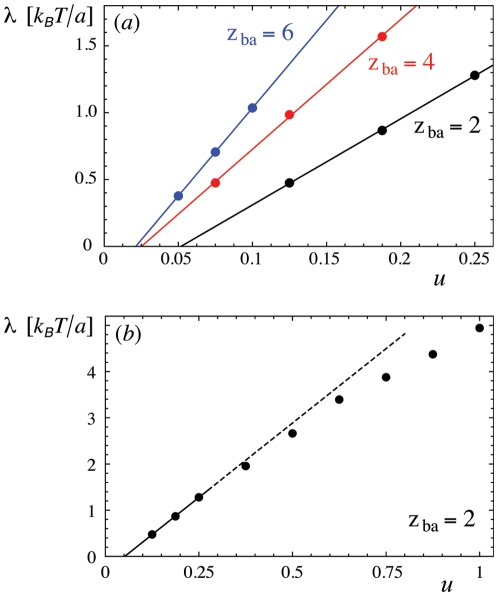
Extrapolated line tensions 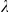 for the symmetric double-well potential with rescaled depth 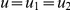 of the potential wells. The data points are from Monte Carlo simulations with the rescaled well width 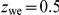 and the rescaled well separations 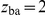, 4 and 6. (a) For small and intermediate values of 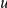, the line tension 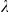 is linear in 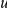; (b) At large values of 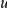, the line tension 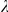 is a nonlinear function of 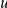. Note that the first three data points are identical with the black data points in subfigure (a). The line tension vanishes at the critical potential depth 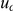 for domain formation, which depends on the separation 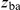 and width 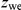 of the two potential wells (see eqs. (14) and (15)).

To derive a general relation for the line tension 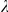, we now focus on the extrapolated line tensions for the symmetric double-well potential. The symmetric double-well potential corresponds to the equilibrium situation in the case of large coexisting domains since the free-energy difference per area (18) between the domains vanishes for equal rescaled well depths 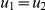 [26], [31]. Our values for the extrapolated line tensions at different rescaled depths 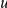 and separations 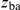 are shown in fig. 6. Each of the data points in this figure results from an extrapolation analogous to fig. 5.

For small and intermediate values of 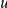, the line tension 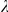 depends linearly on 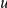 (see fig. 6(a)). This linear dependence is in agreement with previous evidence [35], [36] that the critical point of membranes in a double-well potential is in the same universality class as the critical point of the two-dimensional Ising model. In the vicinity of the critical temperature 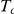, the line tension in the Ising model depends linearly on 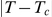 for 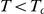. Therefore, the line tension 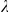 of the membrane domains can be expected to depend linearly on 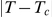 for 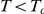 as well, which implies a linear dependence on 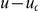 for 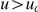 in the vicinity of the critical potential depth 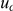.

The critical potential depth 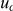 can be estimated from extrapolation to 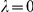 since the line tension 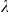 vanishes at the critical point. From the three curves in fig. 6(a), we obtain the values 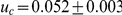, 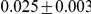, and 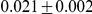 for 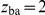, 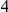, and 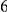. Within the numerical accuracy, these values agree with the values 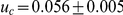, 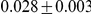, and 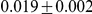 obtained from eq. (15). This agreement confirms our approach since eq. (15) has been derived independently from a finite-size scaling analysis of Monte Carlo data [27].

The values of the rescaled well depth 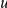 in fig. 6(a) range from 0 to 0.25. For this range of values, the fraction 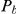 of membrane patches bound in a single well only depends on 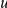, and not on the well width 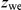 (see fig. 2). We therefore expect that the values of 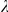 shown in fig. 6(a) only depend on the rescaled depth 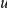 and the ratio 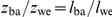 of the separation and width of the wells. The line tension 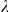 in 6(a) is linear in 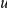 and vanishes at the critical depth 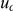. From a dimensional analysis (see Appendix S1), we obtain the scaling form
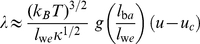
(23)for the line tension in the vicinity of the critical point.

**Figure 7:**
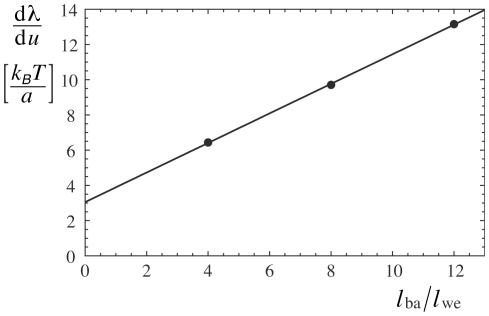
Slopes of the three curves in fig. 6(a) as a function of the ratio 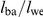 of the separation and width of the potential wells. The slopes can be well fitted by a linear function (see text).

The scaling function 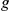 in eq. (23) can be obtained from an analysis of the slope of the three lines in fig. 6(a) as a function of 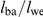 (see fig. 7). From the Monte Carlo simulations, we obtain the line tension in units of 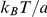. To extract the scaling function 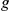 from the Monte Carlo data, we note that eq. (23) can be written as
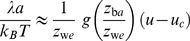
(24)where 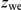 and 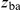 are the rescaled width and separation of the potential wells defined in eq. (10). The three data points in fig. 7 for the slopes of the three lines in fig. 6(a) can be well fitted with a linear function. According to eq. (24), this linear function is 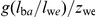. For the rescaled well width 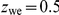 used in our Monte Carlo simulations, we obtain
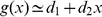
(25)with 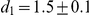 and 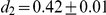. From a previous scaling analysis of 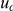 [27], we expect that eq. (23) holds for 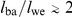. However, the scaling relation (23) is not unreasonable in the limit of small 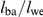. In this limit, the line tension 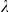 vanishes since 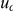 diverges according to eq. (15) and since 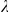 is 0 for 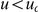.

### Minimum sizes of stable TCR microdomains

We consider now a situation in which a domain of long receptor-ligand complexes 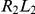 extends over the whole adhesion zone of two cells, and determine the critical size for the nucleation of microdomains of short 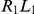 complexes within this large 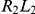 domain. This situation corresponds to a T cell that adheres to a second cell *via* long integrin complexes and that forms microdomains of short TCR-MHCpeptide complexes if foreign MHCpeptides are present on the apposing cell surface. According to classical nucleation theory, the critical radius beyond which these microdomains are stable is 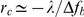 (see eq. (22)). From our general relations (23) and (20) for the line tension 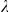 and the free energy difference 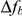, we obtain
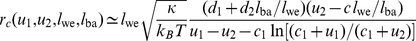
(26)with numerical parameters 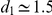, 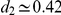 (see eq. (25)), 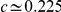 (see eq. (15)), and 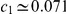 (see eq. (20)). The nucleation of a microdomain of short receptor-ligand complexes 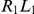 within the large 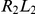 domain can only occur for effective binding energies 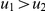 of the domains. We assume here that the line tension for this nucleation event can be estimated by eq. (23) with 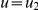 since the barrier crossed in the event has the height 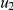.

To estimate the magnitude of the rescaled effective binding energy 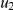 of the domain of 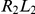 complexes, we assume now the values 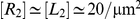 and 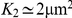 for the concentrations and binding constants of the receptors and ligands, which lead to the effective binding energy 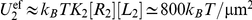 of these complexes (see eq. (8)). The rescaled effective binding energy defined in eq. (13) is then 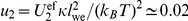 for the interaction range 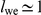 nm of the complexes and the effective bending rigidity 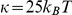 of the membranes. The fraction 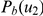 of the membranes within binding range of the receptors and ligands is then approximately 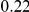 according to eq. (19), and the concentration of bound receptor-ligand complexes is 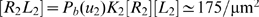 [33]. These concentrations are within the range of typical concentrations in cell adhesion zones [37].

**Figure 8:**
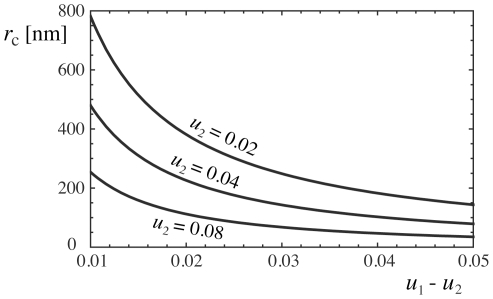
Critical radius 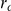 for the nucleation of a microdomain of short 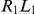 complexes within a large domain of long 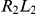 complexes, as a function of the difference 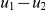 between the rescaled effective binding energies of the domains (see eq. (26)). We have assumed here the values 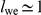 nm and 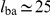 nm for the width and separation of the two wells in the effective potential. The critical radius decreases with 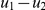 for constant value of 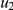, and decreases with 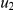 for constant 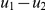.

For T cells, the length difference 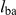 between the TCR-MHCpeptide and the integrin complexes is about 25 nm, which leads to the ratio 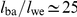 of the separation and width of the two wells in the effective potential. According to eq. (15), the critical rescaled well depth for domain formation is then 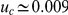. As required for domain coexistence, this value of the critical well depth is below our estimate for 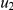, and also below 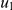 since nucleation of the 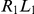 microdomain implies 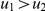. In fig. 8, the critical radii 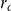 obtained from eq. (26) are plotted as a function of 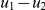. Depending on the difference between 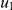 and 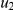, the critical radii vary between tens and hundreds of nanometers, which is in the range of microdomain sizes observed in T-cell adhesion [8], [9], [38], [39].

### Conclusions

While the line tension and stability of lipid domains has been investigated for a long time[40]–[49], the line tension of protein domains in the adhesion zones of membranes has not been addressed, to the best of our knowledge, in previous studies. In this article, we have derived general relations for the line tension 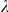 and the free energy difference 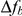 between domains of long and short receptor-ligand complexes in cell adhesion zones (see eqs. (20) and (23)). These relations were obtained from a combination of scaling arguments and Monte Carlo simulations and fully include the thermal shape fluctuations of the membranes. In addition, the degrees of freedom of the receptors and ligands related to their lateral mobility along the membranes are systematically taken into account *via* partial integration in the partition function. These general relations for the line tension and adhesion free energy of the receptor-ligand domains depend only on parameters that can be directly related to experimentally accessible quantities. Using typical values for T-cell adhesion zones, we find that stable submicron-scale domains of TCR-MHCpeptide complexes may form solely because of their length mismatch to integrin complexes. The role of the T-cell cytoskeleton thus may be limited to the observed transport of TCR-MHCpeptide microdomains to the contact zone center *via* weak frictional coupling of the cytoskeleton to the TCRs [50], [51].

## Supporting Information

Appendix S1 **Dimensional analysis.**
(PDF)

Appendix S2 **Free energy of a membrane in a single-well potential.**
(PDF)
