# Supplementary material for: Line Tension and Stability of Domains in Cell-Adhesion Zones Mediated by Long and Short Receptor-Ligand Complexes
Source: PLoS One. 2011 Aug 17;6(8):e23284. doi: 10.1371/journal.pone.0023284 (PMC3157351; doi:10.1371/journal.pone.0023284)
Supplement: Appendix S1 — Dimensional analysis. (PDF) [file pone.0023284.s001.pdf]

Appendix S1: Dimensional analysis

Independent parameters

The starting point of our scaling analysis is the continuous equivalent

$$\mathcal{H}_{\text{ef}} = \iint dx\, dy \left[\frac{\kappa}{2} (\Delta l)^2 + V_{\text{ef}}(l)\right]$$

of the discrete effective conformational energy (6). Here, $\Delta = (\partial^2/\partial x^2) + (\partial^2/\partial y^2)$ is the Laplace operator. The elastic energy depends on the bending rigidity κ, and the effective potential V_{ef} depends on the depths U_1^{ef} and U_2^{ef} as well as the width l_{we} and separation l_{ba} of the two wells (see fig. 1). To reduce the numbers of parameters, we introduce the dimensionless coordinates $\tilde{x}$, $\tilde{y}$ and $\tilde{l}$ defined by

$$x = \tilde{x}\, l_{\text{we}} \sqrt{\frac{\kappa}{k_B T}}\,, \quad y = \tilde{y}\, l_{\text{we}} \sqrt{\frac{\kappa}{k_B T}}\,, \quad l = \tilde{l}\, l_{\text{we}}$$

With these coordinates, we obtain

$$\mathcal{H}_{\text{ef}}/k_B T = \iint d\tilde{x}\, d\tilde{y} \left[\frac{1}{2} \left(\tilde{\Delta}\tilde{l}\right)^2 + \tilde{V}_{\text{ef}}(\tilde{l})\right]$$

with

$$\tilde{\Delta}\tilde{l} = \Delta l \frac{\kappa l_{\text{we}}}{k_B T} \ \text{ and } \ \tilde{V}_{\text{ef}}(\tilde{l}) = \frac{\kappa l_{\text{we}}^2}{(k_B T)^2} V_{\text{ef}}(l)$$

The three dimensionless, independent parameters thus turn out to be

$$u_1 = \frac{U_1^{\text{ef}} \kappa l_{\text{we}}^2}{(k_B T)^2}\,, \quad u_2 = \frac{U_2^{\text{ef}} \kappa l_{\text{we}}^2}{(k_B T)^2}\,, \quad \frac{l_{\text{ba}}}{l_{\text{we}}}$$

Line tension scaling

We consider now a one-dimensional interface, or line, Γ and its energy

$$E_\lambda = \int_\Gamma ds\, \lambda$$

with line tension λ. The integration is performed along the contour Γ that lies in the x-y plane. In analogy to the previous section, we introduce the dimensionless coordinate $\tilde{s}$ defined by

$$s = \tilde{s}\, l_{\mathrm{we}} \sqrt{\frac{\kappa}{k_B T}}$$

The line energy is then

$$E_\lambda = k_B T \int d\tilde{s}\, \tilde{\lambda}$$

with the dimensionless line tension

$$\tilde{\lambda} = \frac{\lambda l_{\mathrm{we}} \kappa^{1/2}}{(k_B T)^{3/2}}$$

In general, $\tilde{\lambda}$ is a function of the three dimensionless parameters u_1, u_2 and $l_{\mathrm{ba}}/l_{\mathrm{we}}$. But for the symmetric double-well potential with $u_1 = u_2 = u$, the dimensionless line tension $\tilde{\lambda}$ depends only on the two parameters u and $l_{\mathrm{ba}}/l_{\mathrm{we}}$. The Monte Carlo results shown in fig. 6 indicate that λ is proportional to $u - u_c$ in the vicinity of the critical point u_c. Close to the critical point, the dimensionless line tension thus can be written in the form

$$\tilde{\lambda} \approx g(l_{\mathrm{ba}}/l_{\mathrm{we}})(u - u_c)$$

with an unknown function g. From this equation, we now obtain the scaling form (23) of the line tension λ.
