# Supplementary material for: Line Tension and Stability of Domains in Cell-Adhesion Zones Mediated by Long and Short Receptor-Ligand Complexes
Source: PLoS One. 2011 Aug 17;6(8):e23284. doi: 10.1371/journal.pone.0023284 (PMC3157351; doi:10.1371/journal.pone.0023284)
Supplement: Appendix S2 — Free energy of a membrane in a single-well potential. (PDF) [file pone.0023284.s002.pdf]

Appendix S2: Free energy of a membrane in a single-well potential

We start from the partition function

$$\mathcal{Z} = \left[\prod_i \int_0^\infty \mathrm{d}l_i\right] e^{-\left(\mathcal{H}_{\rm el}\{l\}+a^2\sum_i V(l_i)\right)/k_BT}$$

of a membrane bound by the single-well potential

$$V(l_i) = -U\theta(l_{\rm we}/2 - |l_i - l_o|)$$

The potential well has the depth U, width $l_{\rm we}$ and is centered at the membrane separation l_o. The derivative of the free energy $\mathcal{F} = -k_BT\ln\mathcal{Z}$ with respect to the depth U of the well can be written as

$$\frac{\partial\mathcal{F}}{\partial U} = -AP_b$$

where $P_b\left(U, l_{\rm we}\right) = \langle\theta(l_{\rm we}/2 - |l_i - l_o|)\rangle$ is the average fraction of membrane sites within the potential well, and $A = \sum_i a^2$ is the total membrane area. The free energy difference between two states with potential depths U_1 and U_2 is therefore

$$\Delta\mathcal{F} = -A\int_{U_1}^{U_2} P_b\left(U, l_{\rm we}\right) dU$$

The scaling analysis of appendix A indicates that P_b depends primarily on the rescaled depth

$$u = Ul_{\rm we}^2\kappa/\left(k_BT\right)^2$$

A change of variables now leads to

$$\Delta f = -\frac{(k_BT)^2}{\kappa l_{\rm we}^2}\int_{u_1}^{u_2} P_b(u)du$$

with $\Delta f = \Delta\mathcal{F}/A$.

The function $P_b(u)$ can be determined from Monte Carlo simulations of a membrane bound in a single well (see fig. 2). We find that the Monte Carlo data can be well described by

$$P_b(u) \simeq P_b^{(3)}(u) = \frac{u + c_2u^2 + c_3u^3}{c_1 + u + c_2u^2 + c_3u^3}$$

with the three fit parameters $c_1 \simeq 0.073$, $c_2 \simeq -0.99$, and $c_3 \simeq 6.44$ for the rescaled width $z_{\text{we}} = 0.5$. For $u \lesssim 0.2$, the three-parameter function $P_b^{(3)}(u)$ for the rescaled width $z_{\text{we}} = 0.5$ coincides with the function $P_b^{(3)}(u)$ for $z_{\text{we}} = 1$ (see full lines in fig. 2), which illustrates that the rescaled well depth u is the dominant parameter in this range. Dimensional analysis indicates that u is the dominant parameter as long as the lateral correlation length of the membrane fluctuations is significantly larger than the discretization length a (see text S1). For strongly bound membranes with values of P_b close to 1, the correlation length is comparable to a, and the precise value of P_b depends also on the rescaled well width z_{we}.
